# Identification of potential biomarkers related to mesenchymal stem cell response in patients with Alzheimer’s disease

**DOI:** 10.1186/s13287-023-03410-8

**Published:** 2023-07-19

**Authors:** Yejoo Choi, Sungho Shin, Hyo Jin Son, Na-Hee Lee, Su Hyeon Myeong, Cheolju Lee, Hyemin Jang, Soo Jin Choi, Hee Jin Kim, Duk L. Na

**Affiliations:** 1 Cell and Gene Therapy Institute (CGTI), Research Institute for Future Medicine, Samsung Medical Center, Seoul, Republic of Korea; 2 Department of Neurology, Samsung Medical Center, Sungkyunkwan University School of Medicine, 81 Irwon-ro, Gangnam-gu, Seoul, 06351 Republic of Korea; 3 Chemical and Biological Integrative Research Center, Korea Institute of Science and Technology, Seoul, 02792 Republic of Korea; 4 School of Medicine, Sungkyunkwan University, 81 Irwon-ro, Gangnam-gu, Seoul, 06351 Republic of Korea; 5 Neuroscience Center, Samsung Medical Center, 81 Irwon-ro, Gangnam-gu, Seoul, 06351 Republic of Korea; 6 Department of Health Sciences and Technology, SAIHST, Sungkyunkwan University, Seoul, 06355 Republic of Korea; 7 Division of Bio-Medical Science and Technology, KIST School, Korea University of Science and Technology, Seoul, 02792 Republic of Korea; 8 Alzheimer’s Disease Convergence Research Center, Samsung Medical Center, Seoul, Republic of Korea; 9 Biomedical Research Institute, MEDIPOST Co., Ltd., 21, Daewangpangyo-ro 644 Beon-gil, Seongnam-si, Gyeonggi-do 13494 Republic of Korea

**Keywords:** Alzheimer’s disease, Mesenchymal stem cell, LC–MS/MS, Biomarker

## Abstract

**Background:**

Preclinical studies showed that mesenchymal stem cells (MSCs) ameliorate tau phosphorylation, amyloid-beta accumulation, and inflammation in Alzheimer’s disease (AD) mouse models via secretion of neurotrophic factors and cytokines. We aimed to identify CSF biomarkers that can be used to predict or monitor the response to MSCs in patients with AD.

**Methods:**

AD patients were injected with human umbilical cord blood-MSCs (*n* = 22) or placebo (*n* = 12). The cerebrospinal fluid (CSF) samples were collected at baseline, one day after the first injection, and one day after the third injection. The patients injected with MSCs were classified into good responder (GR) or poor responder (PR) groups based on the rate of changes in the ratio of total-tau and phosphorylated-tau in the CSF. We selected three typical participants in each group, and their CSF protein levels were analyzed using liquid chromatography/tandem mass spectrometry (LC–MS/MS).

**Results:**

In the LC–MS/MS analysis, 1,667 proteins were identified. Eleven proteins showed significant differences between the typical GR and PR at baseline. Based on their significance level and known functions, two proteins, reticulocalbin-3 (RCN3) and follistatin-related protein 3 (FSTL3), were selected as potential biomarkers to predict MSC response. A total of 173 proteins showed significant change one day after the third injection compared to the baseline in typical GR. We excluded 45 proteins that showed significant change after the third injection compared to the baseline in the typical PR. Based on their significance level and known function, four proteins, scrapie-responsive protein 1 (SCRG1), neural proliferation differentiation and control protein (NPDC1), apolipoprotein E (ApoE), and cystatin C (CysC), were selected as potential biomarker to monitor MSC response. Additionally, functional analysis revealed that the increased CSF proteins after the third injection compared to the baseline in the typical GR were associated with synaptogenesis.

**Conclusions:**

This study identified two proteins (RCN3 and FSTL3) that may be potential biomarkers for predicting MSC response and four proteins (SCRG1, NPDC1, ApoE, CysC) that may be potential biomarkers for monitoring MSC response in patients with AD. Further studies are needed to validate our results.

*Trial registration* Clinical Trials.gov, NCT02054208. Registered on 4 February 2014. Samsung Medical Center IRB File No.2017-04-025. Registered on 20 June 2017.

**Supplementary Information:**

The online version contains supplementary material available at 10.1186/s13287-023-03410-8.

## Background

Alzheimer’s disease (AD) is the most common neurodegenerative disease. The formation of amyloid-beta (Aβ) plaques and neurofibrillary tangles by abnormally phosphorylated tau is pathological hallmarks of AD [1, 2]. Multiple pathways are involved in the pathogenesis of Aβ and tau accumulation [1, 3–5]. Therefore, therapeutics that target multiple pathways are necessary to ameliorate AD pathology.

Mesenchymal stem cells (MSCs) are multipotent stem cells that can self-renew and differentiate. When administered to the brain, MSCs perform paracrine action by secreting various cytokines and chemokines [6, 7] that contribute to neuroprotection [8, 9]. In AD animal models, MSCs improved neurogenesis, memory deficits, synaptic dysfunction, and neuroinflammation [10–14]. Previous research has shown that MSCs have the potential to multitarget numerous pathomechanisms involved in AD pathogenesis.

Clinical trials on MSC transplantation have been conducted in various diseases [15–20]. In AD, phase 1 clinical trials have shown that human umbilical cord blood-derived MSCs (hUCB-MSCs) administered to the parenchyma (hippocampus and precuneus) or lateral ventricle were safe and tolerable [21, 22]. A recently completed phase IIa double-blind randomized clinical trial (ClinicalTrials.gov, NCT02054208) showed that although MSCs did not improve clinical symptoms, the decrement of total tau (T-tau) and phosphorylated-tau (P-tau) in the cerebrospinal fluid (CSF) was greater in patients administrated with MSCs in the lateral ventricle compared to the placebo group [23]. However, biomarkers for predicting or monitoring MSC effects have not yet been identified.

In this study, we aimed to identify CSF biomarkers that can be used to predict or monitor the response to MSCs when administered into the ventricular space in AD patients. We used liquid chromatography/tandem mass spectrometry (LC–MS/MS) to analyze CSF protein levels in three patients who showed good response to MSCs (Good responder, GR), three patients who showed poor response to MSCs (Poor responder, PR), and three patients who were administered with placebo. GR and PR were categorized based on the rate of change in CSF T-tau and P-tau levels one day after the third injection compared to that at the baseline.

## Materials and methods

### Participants and collection of CSF

We collected CSF samples from patients with AD who participated in a phase IIa clinical trial (Safety and Exploratory Efficacy Study of NEUROSTEM® Versus Placebo in Patients with Alzheimer's Disease, Clinical Trials.gov, NCT02054208). In this clinical trial, either 3.0 × 10^7^ MSCs/2 ml (*n* = 24) or placebo (*n* = 12) was administered into the lateral ventricle thrice at 4-week intervals via the Ommaya reservoir [22]. hUCB-MSC manufacture, cell quality control, and quality assurance were performed at MEDIPOST (see Additional file 1) [22]. CSF samples were collected at baseline, one day after the first injection, and one day after the third injection. The CSF was obtained via the Ommaya reservoir and aliquoted into 1 mL polypropylene tubes and stored in a deep freezer at − 80 °C.

The participants were diagnosed with AD dementia or mild cognitive impairment due to AD, as suggested by the National Institute on Aging-Association Alzheimer's workgroup (NIA-AA) [24, 25]. All participants were positive for florbetaben positron emission tomography (PET). CSF samples from two participants among the 36 were not available. Thus, in this study, we analyzed CSF proteins from 34 participants. All participants provided written informed consent, and the study was approved by the Institutional Review Board of Samsung Medical Center (IRB File No.2017-04-025).

### Classification of GR and PR in the hUCB-MSC administration group

**Fig. 1:**
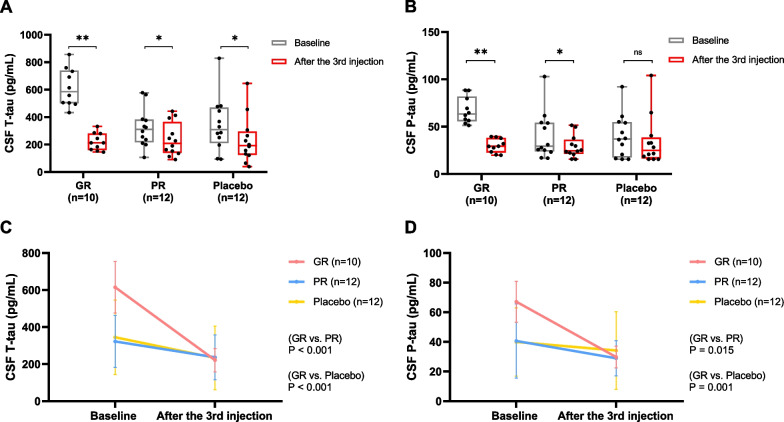
CSF total-tau and phosphorylated-tau levels. CSF (**A**) T-tau and (**B**) P-tau levels at baseline and one day after the third MSC injection in each group. Intergroup comparison of rate of changes in CSF (**C**) T-tau and (**D**) P-tau levels. In the Mixed effects model, we included time, group, baseline T-tau or P-tau levels, and time*group as fixed effect and subjects as random effects. *p < 0.05, **p < 0.001. *GR* Good Responder; *PR* Poor Responder; *CSF* Cerebrospinal Fluid; *T-tau* Total-tau; *P-tau* Phosphorylated-tau

Participants in the hUCB-MSC administration group (*n* = 22) were categorized into GR or PR based on their response to MSCs. The response was determined by the rate of change in CSF T-tau and P-tau (P-tau 181) levels one day after the third injection compared to that at the baseline. We defined GR as follows: (T-tau level at one day after the third injection / T-tau level at baseline) ≤ 0.7 and (P-tau level at one day after the third injection/P-tau level at baseline) ≤ 0.7. Ten participants were classified as GR and 12 were classified as PR. The T-tau and P-tau levels in each group are shown in Fig. 1A, B, respectively. Mixed effects model showed that CSF T-tau levels decreased more rapidly in the GR than in the PR (*p* < 0.001) or placebo (*p* < 0.001) group even after controlling for baseline T-tau level (Fig. 1C). Furthermore, mixed effects model showed that CSF P-tau levels decreased more rapidly in the GR than in the PR (*p* = 0.015) or placebo (*p* = 0.001) group even after controlling for baseline P-tau level (Fig. 1D).

Three typical participants from each group were selected for LC–MS/MS examination. Among the GR group, we selected three typical GR participants who showed the most decrease in CSF T-tau and P-tau levels after the third injection compared to that at the baseline levels. Likewise, among the PR group, we selected three typical PR participants who showed the least decrease in CSF T-tau and P-tau levels after the third injection compared to that at the baseline levels. In the placebo group, we selected three participants who were matched in age and baseline CSF tau levels (T-tau: 331.816–613.608 pg/mL, P-tau: 53.768–62.041 pg/mL) to the three typical GR and three typical PR participants.

### CSF sample preparation for LC–MS/MS

CSF contains high concentrations of proteins that interfere with mass spectrometry, such as albumin and immunoglobulins [26]. The top 14 abundant proteins were depleted using a MARS14 column (Agilent, CA, USA). For this, the mixture was diluted 1:1 with a proprietary “Buffer A” and loaded onto a MARS14 column on an Agilent 1100 series HPLC system (Agilent). The unbound fraction was buffer exchanged into 8 M urea in 50 mM Tris (pH 8) and concentrated through ultrafiltration using an Amicon Ultra-0.5 mL 3 kDa cutoff filter (Millipore, Darmstadt, Germany) to approximately 50 μL. Samples were treated with 5 µL of 50 mM tris (2-carboxyethyl) phosphine at 25 °C for 1 h and further treated with 5 µL of 150 mM iodoacetamide at 25 °C for 1 h in the dark. Urea concentration was diluted to 0.8 M with 50 mM Tris prior to trypsin digestion (Promega, Madison, WI, USA) with an enzyme to substrate ratio of 1:50 and incubated at 37 °C for 16 h on a shaker at 600 rpm. Formic acid was then added at a final concentration of 0.2% to stop the digestion reaction. The peptides were desalted using an Oasis HLB 1 cc Vac cartridge (Waters, Milford, MA, USA) and dried with a vacuum centrifuge (miVac Duo Concentrator; Genevac, Suffolk, UK). The dried peptides were resuspended in labeling buffer (0.1 M triethylammonium bicarbonate buffer, Sigma-Aldrich), and the peptide concentration was determined using the quantitative colorimetric peptide assay (Thermo Fisher Scientific, Waltham, MA, USA). An aliquot equivalent to 25 μg (100 μL) of each sample was immediately labeled with 0.8 mg of each tandem mass tag (TMT) channel, except for TMT 126, which was prepared according to the manufacturer’s instructions. The control sample was labeled with TMT channel 126 for normalization. Following incubation at room temperature for 1 h, the reaction was quenched with hydroxylamine at a final concentration of 0.3% (v/v). The TMT-labeled samples were combined and dried *in vacuo*.

### Peptide fractionation and preparation of proteome samples

The TMT-labeled peptides were fractionated by basic pH reversed-phase liquid chromatography using an Agilent 1290 Infinity LC System (Agilent). Chromatography was performed using an XBridge BEH130 C18 column (4.6-μm i.d. × 250 mm length; pore size of 130 Å and particle size of 3.5 μm; Waters Corporation, Milford, MA, USA) at a flow rate of 0.5 mL/min. The mobile phases were 10 mM NH4HCO2 (pH 10) as phase A and 10 mM NH4HCO2 (pH 10) in 90% ACN (pH 10) as phase B. The peptides were dissolved in 110 μL of mobile phase A and then, injected into a 100-μL sample loop. The gradient was 5% B for 10 min, 5–40% B for 40 min, 40–70% B for 15 min, 70% B for 10 min, and 70–5% B for 15 min. Fractionation was performed by collecting 84 tubes (0.8 min/tube) throughout the chromatographic run. Eighty-four fractions were pooled to obtain 12 concatenated fractions and dried in vacuo.

### LC–MS/MS

The dried peptide samples were reconstituted in 0.2% acetic acid, and an aliquot containing ~ 1 μg of the sample was injected from a cooled (10 °C) autosampler into a reversed-phase Magic C18aq (Michrom Bioresources, Auburn, CA, USA) column (20 cm × 75 μm, packed in-house) on an Eksigent nanoLC-ultra 1D plus system at a flow rate of 300 nL/min. Before use, the column was equilibrated with 95% buffer A (0.1% formic acid in water) and 5% buffer B (0.1% formic acid in 80% acetonitrile). The peptides were eluted with a linear gradient from 5–40% buffer B over 140 min, 40–95% buffer B over 2 min and then, subjected to an organic wash and aqueous re-equilibration at a flow rate of 300 nL/min with a total run time of 200 min. The HPLC system was coupled to a Q-Exactive HF-X mass spectrometer (Thermo Fisher Scientific) operated in data-dependent acquisition mode. Survey full-scan MS spectra (m/z 350–1500) were acquired at a resolution of 120,000. The source ionization parameters were as follows: spray voltage, 2.5 kV; capillary temperature, 300 °C; and s-lens level, 44.0. The MS/MS spectra of the 20 most intense ions from the MS1 scan with a charge state of 2–5 were acquired with a fixed first m/z of 110 along with the following options: resolution, 45,500; automatic gain control target, 1E5; isolation width, 0.7 m/z; normalized collision energy, 32%; dynamic exclusion duration, 40 s; and ion selection threshold, 1 × 10^5^ counts.

### Analysis of mass spectrometric data (peptide and protein identification and quantification)

The mass spectrometric data were loaded onto the Proteome Discoverer (version 2.2.0.388) software. Protein identification was performed using the Human UniProtKB database (released in June 2020). The integration of reporter ion tolerance was selected as 20 ppm, precursor mass tolerance was 10 ppm, and the fragment mass tolerance was 0.02 Da. Search parameters included two missed trypsin cleavage sites, cysteine carbamidomethylation (+ 57.021 Da), and lysine TMT-label (+ 229.163) as fixed modifications, methionine oxidation (+ 15.9949 Da) and N-terminal protein acetylation (+ 42.0106 Da) as variable modifications. The false discovery rate (FDR) was set to 0.01 for proteins and peptides and was determined by searching a reverse database. For protein identification, one peptide was required as the minimum number of razor and unique peptides in a protein group.

### Candidate proteins to predict and monitor MSC response

To predict the response to MSC administration in patients with AD, we selected the proteins that were significantly different between typical GR and typical PR at baseline using student’s t-test (FDR *q*-value < 0.05, $\left|{\mathrm{log}}_{2}Fold Change (FC)\right|$ ≥ 1.5, unique peptide ≥ 2). FC for prediction biomarkers was calculated by baseline protein levels in typical GR / baseline protein levels in typical PR.

To monitor the effectiveness of MSC administration, we selected the proteins whose expressions changed significantly after the third MSC injection compared to that at the baseline in the typical GR using Student’s t-test (FDR *q*-value < 0.05, $\left|{\mathrm{log}}_{2}FC\right|$ ≥ 1.8, unique peptide ≥ 2). FC for monitoring biomarkers was calculated by protein levels at one day after the third injection in typical GR / protein levels at baseline in typical GR. Next, to select the proteins whose expressions were significantly changed only in the typical GR, we excluded proteins that showed a significant change after the third MSC injection compared to that at the baseline in the typical PR (FDR *q*-value < 0.05).

### Enzyme-linked immunosorbent assay (ELISA)

To validate the potential biomarkers identified in LC–MS/MS, we performed ELISA in all participants (*n* = 34). Reticulocalbin (RCN3) levels were measured using Human Reticulocalbin 3 (RCN3) ELISA kit (Abbexa LTD, Cambridge, UK). Follistatin-related protein 3 (FSTL3) and Cystatin-C (CysC) levels were measured using DuoSet ELISA kit (catalog number: DY1288B and DY1196, R&D systems Minneapolis, MN, USA). Scrapie-responsive protein 1 (SCRG1) levels were measured using Human SCRG1 ELISA kit (BIOMATIK, Wilmington, Delaware, USA). Neural proliferation differentiation and control protein 1 (NPDC1) levels were measured using Human NPDC1 ELISA Kit (RayBiotech, USA). Apolipoprotein E (ApoE) levels were measured using Human APOE ELISA Kit (abcam, Cambridge, UK). All the samples were measured in triplicate. The experiments were conducted according to the manufacturer’s instructions.

### Functional annotation and canonical pathway analysis

Functional annotation analysis was performed to evaluate the function of the CSF proteins identified significantly for each group. The functional annotation tool Database for Annotation, Visualization and Integrated Discovery (DAVID) version 6.8 was used, and the protein data set was uploaded based on the UniProt Accession number [27].

In the gene ontology analysis, the annotation was categorized with regard to biological processes, cellular components, and molecular functions. Each categorization utilized a “GOTERM DIRECT” default setting. All gene ontologies included in biological processes, cellular components, and molecular functions met the following criteria: threshold count ≥ 2, EASE (*p*-value) ≤ 0.01, and Benjamini (FDR *q*-value) < 0.05. Fold enrichment was automatically computed using the DAVID program. The prediction of canonical pathway analysis was performed using the IPA program (Qiagen, Venlo, Netherlands). For canonical pathway analysis, proteins with FDR *q*-values < 0.05 were used in the results.

### Statistical analysis

To test the difference in CSF protein levels between the baseline and after the third injection, paired t-test and Wilcoxon matched-pairs signed-rank test were used. To test the difference in the change of CSF T-tau and P-tau levels between the groups, mixed effects model was used. Time, group, baseline T-tau or P-tau levels were included as fixed effects, and subject was included as a random effect.

In LC–MS/MS analyses, the protein intensity of a sample was calculated in reference to the control sample (TMT-126) and converted to the log2 scale. Perseus (version 1.5.8.4) [28] was used for all other bioinformatic analyses performed in this study. All quantified proteins were width adjustment normalized across the stage. In ELISA analyses, we used student’s t-test or paired t-test (one-tailed) to validate the potential biomarkers identified in the LC–MS/MS. Statistical significance was set at *p* < 0.05. Statistical analyses were performed using GraphPad Prism version 8.0, and IBM SPSS statistics 21.

## Results

### Characteristics of typical participants in each group

The GR group had higher levels of baseline CSF Aβ (*p* = 0.007), T-tau (*p* < 0.001), and P-tau (*p* = 0.023) levels compared to that in the PR group. Although it did not reach significance level, the GR group tended to have a lower Alzheimer’s Disease Assessment Scale-Cognitive Subscale score (ADAS-Cog) than the PR group (*p* = 0.054), indicating better cognitive function at baseline (Table 1).

**Table 1:** The demographic data of GR, PR, and Placebo group

	Placebo (n = 12)	GR (n = 10)	PR (*n* = 12)	*p*-value Placebo vs. GR	*p*-value Placebo vs. PR	*p*-value GR vs. PR
Age	61.8 ± 5.01	65.8 ± 7.11	65.1 ± 6.72	0.429	0.613	> 0.999
Sex(Female)	6 (50.0%)	9 (90.0%)	8 (66.7%)	0.074	0.680	0.323
*APOE* ε4 carrier	7 (58.3%)	8 (80.0%)	5/11 (45.5%)†	0.381	0.684	0.183
Baseline amyloid beta(pg/mL), mean ± SD	202.47 ± 112.38	431.53 ± 153.55	242.68 ± 132.93	0.001*	> 0.999	0.007*
Baseline T-Tau(pg/mL), mean ± SD	344.80 ± 200.80	614.99 ± 139.08	322.06 ± 140.61	0.002*	> 0.999	< 0.001*
Baseline P-tau(pg/mL), mean ± SD	39.94 ± 23.02	67.10 ± 13.90	40.63 ± 25.05	0.019*	> 0.999	0.023*
ADAS-Cog, mean ± SD	20.3 ± 4.44	18.6 ± 4.77	24.1 ± 5.98	> 0.999	0.248	0.054
MMSE, mean ± SD	22.5 ± 4.12	23.9 ± 3.81	20.4 ± 4.36	> 0.999	0.675	0.172
Florbetaben PET positive	12 (100%)	10 (100%)	12 (100%)	> 0.999	> 0.999	> 0.999

^†^One participants in PR didn’t have available APOE genotype data. *GR* Good Responder; *PR* Poor Responder; *ADAS-Cog* Alzheimer’s Disease Assessment Scale-Cognitive Subscale; *MMSE* Mini-Mental State Examination; *SD* Standard Deviation; *PET* Positron Emission Tomography

*p < 0.05

We selected three typical participants each from the GR and PR groups based on the changes in CSF T-tau and P-tau for LC–MS/MS analysis. The fold changes in CSF T-tau and P-tau after the third MSC administration compared to the baseline were calculated. Three participants who showed the lowest CSF T-tau and P-tau fold change were chosen as typical GR. CSF T-tau levels of GR1, GR2, and GR3 decreased to 0.27-, 0.21-, and 0.35-fold, and their CSF P-tau levels decreased to 0.38-, 0.24-, and 0.31-fold, respectively, after the third injection as compared to the baseline levels. Meanwhile, three participants who showed the highest CSF T-tau and P-tau fold change were chosen as typical PR. In the typical PR, CSF T-tau levels of PR1, PR2, and PR3 increased to 1.45-, 1.18-, and 1.19- fold, respectively, after the third injection as compared to the baseline levels. CSF P-tau levels of PR1, and PR2 increased to 1.31- and 1.17- fold, respectively, and the P-tau level of PR3 decreased to 0.96-fold after the third injection as compared to the baseline levels. We selected three placebo participants by matching the age, baseline CSF T-tau and P-tau levels of the typical GR and typical PR. CSF T-tau levels of Placebo1 increased to 1.34-fold and the level of Placebo2 and Placebo3 decreased to 0.60-, and 0.43-fold, respectively, after the third normal saline injection as compared to the baseline levels. CSF P-tau levels of Placebo1 increased to 1.71-fold and that of Placebo2 and Placebo3 decreased to 0.75- and 0.51-fold, respectively, after the third normal saline injection as compared to the baseline. Placebo1, Placebo2, and Placebo3 had baseline CSF T-tau levels of 481.486, 444.257, and 367.649 pg/mL, respectively. Their baseline CSF P-tau levels were 60.822 pg/mL, 54.097 pg/mL, and 55.11 pg/mL, respectively.

The clinical characteristics of typical participants are shown in Table 2. MRIs of the nine participants revealed no evidence of moderate to severe white matter hyperintensities. All participants were florbetaben-PET positive.

**Table 2:** Clinical characteristics of nine typical participants

Group	Age	Sex	ADAS-Cog	MMSE	Amyloid PET
GR1	60	F	18	25	Positive
GR2	61	F	13	29	Positive
GR3	67	F	22	22	Positive
PR1	60	F	24	25	Positive
PR2	65	F	19	24	Positive
PR3	58	F	20	25	Positive
Placebo1	71	M	21	26	Positive
Placebo2	64	F	24	19	Positive
Placebo3	56	F	26	18	Positive

*GR* Good Responder; *PR* Poor Responder; *ADAS-Cog* Alzheimer’s Disease Assessment Scale-Cognitive Subscale; *MMSE* Mini-Mental State Exam

### Identification of proteins from LC–MS/MS analysis

**Fig. 2:**
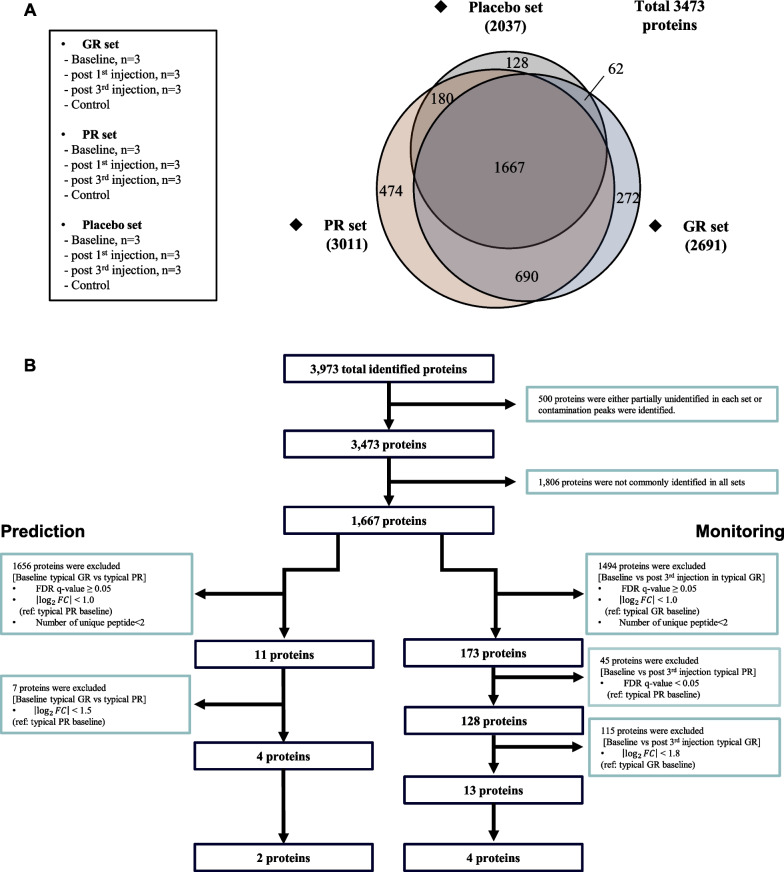
Identification of proteins to predict and monitor MSC response using LC–MS/MS. **A** The set composition for liquid chromatography/tandem mass spectrometry analysis (LC–MS/MS) and Venn diagram of identified proteins. 1,667 proteins were commonly identified in three sets. **B** The procedure of narrowing down the candidate proteins. *FDR* False Discovery Rate; *GR* Good Responder; *PR* Poor Responder; *FC* Fold Change

Nine TMT channels in each of the three experiments were classified into three sets: GR, PR, and Placebo (Fig. 2A). After excluding proteins that were partially identified in each set or showed contamination peaks, a total of 3,473 proteins were identified. In the GR set, 2,691 proteins were identified. In the PR set, 3,011 proteins were identified. In the Placebo set, 2,037 proteins were identified. A total of 1,667 proteins were commonly identified in the three sets (Fig. 2A).

### Identifying candidate proteins for prediction or monitoring biomarkers

To search for proteins that can predict MSC response, we compared the baseline CSF protein levels of the typical GR to those of the typical PR (Fig. 2B). Among the 1,667 proteins, we selected 11 proteins that were more than twofold higher or lower ($\left|{\mathrm{log}}_{2}FC\right|$ ≥ 1) in the baseline CSF of the typical GR compared to those of the typical PR with significance of FDR *q* < 0.05 and contained two or more unique peptides. To further narrow the candidate proteins, we selected four proteins that showed $\left|{\mathrm{log}}_{2}FC\right|$ ≥ 1.5 between typical GR and typical PR at baseline. The four proteins were reticulocalbin-3 (RCN3), follistatin-related protein 3 (FSTL3), keratin-type II cytoskeletal, and splicing factor 3A subunit 1 (Table 3, Additional file 2: Figure S1A). Finally, two potential proteins (RCN3 and FSTL3) were chosen for potential prediction biomarkers based on their significance levels and known functions.

**Table 3:** Candidate proteins to predict or monitor MSC response

Category	Protein	Symbol	log_2_FC	FDR *q*-value	Function	References
Prediction biomarkers	Reticulocalbin-3*	RCN3	− 3.21	0.026	Calcium-binding protein	[29]
	Keratin, type 2 cytoskeletal 74	KRT74	− 3.16	0.045	Hair formation	[30]
	Splicing factor 3A subunit 1	SF3A1	− 2.37	0.026	Spliceosome assembly; Pre-mRNA splicing	[31, 32]
	Follistatin-related protein 3*	FSTL3	− 1.70	0.035	Antagonist of transforming growth factor β family; Bind with activin A, and activin B	[33]
Monitoring biomarkers	Bisphosphoglycerate mutase	BPGM	2.96	0.007	Regulate hemoglobin oxygen affinity; Synthesize 2,3-Bisphosphoglycerate	[34, 35]
	Scrapie-responsive protein 1*	SCRG1	2.49	< 0.001	Central nerve system protein; SCRG1 secreted from bone-marrow derived MSCs plays anti-inflammatory role	[36, 37]
	Neural proliferation differentiation and control protein 1*	NPDC1	2.38	0.021	Control neural cell proliferation and differentiation by regulating E2F1 transcription factor	[38]
	Immunoglobulin J chain	JCHAIN	2.21	0.024	Protein component of the antibodies Immunoglobulin M and Immunoglobulin A	[39]
	Dihydropteridine reductase	QDPR	2.18	0.013	Enzyme that catalyzes the recycling of tetrahydrobiopterin	[40]
	Lymphocyte antigen 6H	LY6H	2.09	0.005	Modulate nicotinic acetylcholine receptors activity	[41]
	Cytokine-like protein 1	C17	2.05	0.016	Chondrogenesis, cartilage homeostasis, and osteoarthritis progression	[42]
	Apolipoprotein E*	ApoE	2.02	0.006	Transport Lipids, vitamin, and cholesterol; Bind with amyloid-beta	[43, 44]
	Alpha-L-iduronidase	IDUA	1.96	0.006	Lysosomal degradation of glycosaminoglycans	[45]
	Cystatin-C*	CysC	1.89	< 0.001	Cysteine protease inhibitor; Reduce amyloid-beta aggregation and deposition	[46, 47]
	Aspartate aminotransferase	GOT1	1.86	0.006	Synthesize L-glutamate; Regulate glutamate level	[48]
	Transcobalamin-2	TCN2	1.85	0.006	Transport vitamin B12 to the tissues	[49]
	Divergent protein kinase domain 2B	DIPK2B	1.81	0.009	Few functions are known	–

*Final selection of potential proteins to predict or monitor MSC response. FDR, False Discovery Rate; MSCs, Mesenchymal Stem Cells

Fold change (FC) for prediction biomarkers = baseline protein level in typical GR/baseline protein level in typical PR

FC for monitoring biomarkers = protein level at one day after the third injection in typical GR/protein level in typical GR at baseline

To search for proteins that can monitor MSC response, we compared CSF protein levels after the third MSC injection compared to those at baseline in typical GR. In the typical GR, there was no significant difference in protein levels between the baseline and after the first MSC injection. Of the 1,667 proteins, we selected 173 proteins that were more than twofold increased or decreased ($\left|{\mathrm{log}}_{2}FC\right|$ ≥ 1) in CSF after the third MSC injection with significance of FDR *q* < 0.05 in typical GR, and contained two or more unique peptides. To select proteins that changed significantly only in typical GR, we excluded 45 proteins that showed significant change (FDR *q* < 0.05) after the third injection in typical PR. In typical placebo, there was no significant difference in protein levels between the baseline and after the third normal saline injection. To further narrow the candidate proteins, we selected 13 proteins that showed $\left|{\mathrm{log}}_{2}FC\right|$ ≥ 1.8 after the third MSC injection in the typical GR. The 13 proteins were bisphosphoglycerate mutase, SCRG1, NPDC1, immunoglobulin J chain, dihydropteridine reductase, lymphocyte antigen 6H, cytokine-like protein 1, ApoE, alpha-L-iduronidase, CysC, aspartate aminotransferase, transcobalamin-2, and divergent protein kinase domain 2B (Table 3, Additional file 2: Figure S1B). Finally, four potential proteins (SCRG1, NPDC1, ApoE, and CysC) were chosen for potential monitoring biomarkers based on their significance levels and known functions.

### Potential biomarkers for predicting MSC response

Among the four candidate proteins, RCN3 and FSTL3 were chosen as potential biomarkers for predicting MSC responses according to significance levels and known functions (Table 3, Additional file 2: Figure S1A). RCN3 acts as calcium binding protein [29]. RCN3 levels was lower in the typical GR by 9.26-fold (log_2_FC =  − 3.21) compared to typical PR at baseline (FDR *q* = 0.026). FSTL3, also known as FLRG, is an antagonist of the transforming growth factor β (TGF-β) family [33]. FSTL3 levels was lower in the typical GR by 3.24-fold (log_2_FC =  − 1.70) compared to typical PR at baseline (FDR *q* = 0.035).

### Potential biomarkers for monitoring MSC response

Among the 13 candidate proteins, SCRG1, NPDC1, ApoE, and CysC were selected as potential biomarkers for monitoring MSC responses according to significance levels and known functions (Table 3, Additional file 2: Figure S1B). SCRG1, which is a central nervous system protein, increased by 5.61-fold (log_2_FC = 2.49) after the third MSC injection compared to that at the baseline in the typical GR (FDR *q* < 0.001). NPDC1 is involved in the regulation of cell proliferation and differentiation [38]. After MSC administration, NPDC1 increased by 5.21-fold (log_2_FC = 2.38) (FDR *q* = 0.021). ApoE is a critical protein for lipid metabolism, and the *APOE* ε4 allele increases the risk of AD [50]. ApoE increased by 4.07-fold (log_2_FC = 2.02) after the third MSC injection (FDR *q* = 0.006). CysC is a cysteine protease inhibitor. CysC increased by 3.72-fold (log_2_FC = 1.89) after the third MSC injection (FDR *q* < 0.001).

### Validation of potential biomarkers using ELISA

We further performed ELISA in all participants (*n* = 34) to validate the potential biomarkers identified in the LC–MS/MS. RCN3, one of the potential prediction biomarkers, and SCRG1, one of the potential monitoring biomarkers, were not detected by ELISA. FSTL3, one of the potential prediction biomarkers, did not show significant difference between GR and PR at baseline (Additional file 2: Figure S2A). NPDC1, ApoE, and CysC, the potential monitoring biomarkers, did not show significant increase after the third MSC injection compared to the baseline (Additional file 2: Figure S2B-2D).

### MSC administration up-regulates synaptogenesis in patients with AD

**Fig. 3:**
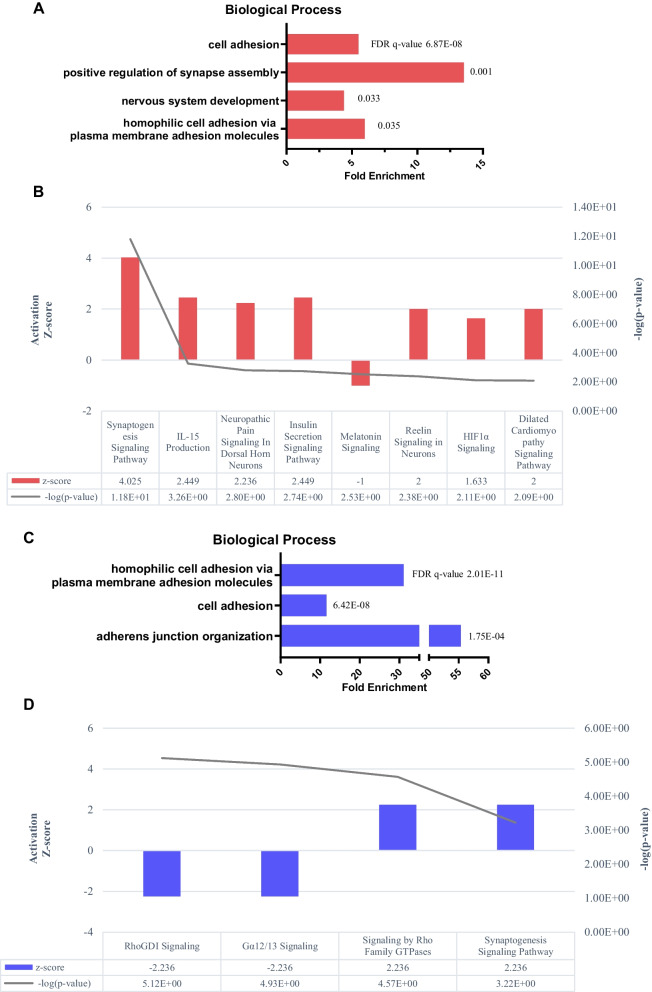
Functional analysis of proteins that showed significant change in the typical GR or PR. **A**, **B** 173 proteins that were significantly increased after the third injection compared to that at the baseline in the typical GR were analyzed. **A** The functional annotation classified in biological processes. **B** Activated canonical pathway after the third injection. **C**, **D** 45 proteins that were significantly increased after the third injection compared to that at baseline in the typical PR were analyzed. **C** The functional annotation classified in biological process. **D** Activated and inactivated canonical pathway after the third injection. Each gene ontology was considered to be significant when FDR *q* < 0.05. The canonical pathway was considered to be significant when − log (*p*-value) ≥ 2.0 and activation Z-score ≥ 2.0 or activation Z-score ≤  − 2.0. *FDR* False Discovery Rate; *GR* Good Responder; *PR* Poor Responder

Gene functional annotation analysis was performed on 173 proteins that increased significantly in the CSF of typical GR after the third injection. A total of 173 genes that encode corresponding proteins were categorized with regard to biological processes, cellular components, or molecular function (Additional file 3: Table S1) based on their function and location. In the biological process, we found that four gene ontologies related to cell adhesion, positive regulation of synapse assembly, nervous system development, and homophilic cell adhesion via plasma membrane adhesion molecules were significantly enriched (FDR *q* < 0.05) (Fig. 3A).

Canonical pathway analysis showed that the proteins increased significantly in the typical GR involved in activating the synaptogenesis signaling pathway with a z-score of 4.025 (Fig. 3B, Additional file 3: Table S1). Additionally, pathways involved in interleukin (IL)-15 production, neuropathic pain signaling in dorsal horn neurons, and the insulin secretion signaling were activated with a high probability (activation Z-score ≥ 2) (Fig. 3B).

Gene functional annotation analysis was performed on 45 proteins whose CSF level increased significantly in the typical PR after the third administration. 45 genes that encode corresponding proteins were categorized with regard to biological processes, cellular components, or molecular function (Additional file 3: Table S1.) based on their function and location. In the biological process, we found that three gene ontologies related to cell adhesion, homophilic cell adhesion via plasma membrane adhesion molecules, and adherens junction organization were significantly enriched (Fig. 3C).

The proteins that were increased significantly following MSC administration in the typical PR were also activated in the synaptogenesis signaling pathway with a Z-score of 2.236 (Fig. 3D, Additional file 3: Table S1). However, the Z-score was lower than that of the typical GR. Additionally, signaling by Rho family GTPases was activated, whereas Rho GDI signaling and the Gα 12/13 signaling pathways were inactivated.

## Discussion

In this study, we aimed to identify CSF biomarkers that can be used to predict or monitor the response to MSC administration in patients with AD. Using LC–MS/MS analysis, we identified two proteins (RCN3, and FSTL3) as potential MSC response prediction biomarkers and four proteins (SCRG1, NPDC1, ApoE, CysC) as potential MSC response monitoring biomarkers. Additionally, functional analysis revealed that the CSF proteins that significantly increased in the typical GR after the third injection were associated with the synaptogenesis. This suggests that increased proteins after MSC administration upregulate the synaptogenesis signaling pathway.

Using LC–MS/MS analysis, we selected RCN3 and FSTL3 as potential biomarkers for predicting MSC response. Low protein levels of RCN3 and FSTL3 at baseline CSF may predict a good response to MSC administration in patients with AD. RCN3 is a calcium-binding protein. Calcium-binding proteins have been implicated in both neuroprotective and neurotoxic responses [29, 51]. Our results support that increased RCN3 at baseline might play a detrimental role in improving AD biomarker levels in response to MSC administration. However, determining whether RCN3 is an appropriate biomarker requires additional functional research. FSTL3 is a TGF-β family antagonist that inhibits activin A and activin B function by binding to activin [33, 52]. As an anti-inflammatory factor in neurodegeneration, activin A plays a critical role in neurogenesis in the adult central nervous system [53]. It has been shown that activin A, originating from MSCs, induces neurodevelopment in the 5XFAD mouse AD model [10]. Therefore, it is probable to suggest the possibility that the high level of FSTL3 at baseline may inhibit activin A secreted from MSCs, resulting in insufficient performance in neurogenesis leading to poor response to MSC administration. We also note that the GR group had higher baseline CSF P-tau and T-tau level compared to the PR group. However, further analyses of mixed effects model showed that the differential responses of T-tau or P-tau level after MSC injection were regardless of baseline T-tau or P-tau levels. Our results may help select participants for future AD MSC clinical trials for AD.

Using LC–MS/MS analysis, we selected SCRG1, NPDC1, ApoE, and CysC as potential monitoring biomarkers for MSC response, which were increased in the typical GR after the third MSC injection compared to that in the baseline. Increased SCRG1, NPDC1, ApoE, and CysC levels in CSF after MSC administration may indicate a good response in patients with AD. SCRG1 is highly expressed in the central nervous system, and SCRG1 secreted by bone marrow-derived MSCs sustains their stemness and anti-inflammatory effects [36, 37]. However, SCRG1 has also been implicated in neuronal autophagy in the transmissible spongiform encephalomyelitis mouse model [54, 55]. Further research on the function of SCRG1 protein in AD is required. NPDC1 is highly expressed in the hippocampus, frontal lobe, and temporal lobe of the adult brain. NPDC1 downregulates cell proliferation and differentiation by regulating the cell cycle by inhibiting E2F-1 transcription factor activity [38]. Neuronal apoptosis is reduced when the E2F-1 transcription factor is suppressed [56]. Therefore, the increased expression of NPDC1 after MSC administration may play a role in inhibiting neuronal cell apoptosis. ApoE, expressed by astrocytes in the brain, plays an important role in lipid transport and metabolism [43]. ApoE binds to Aβ to activate endocytosis and promotes ApoE to uptake Aβ by microglia and astrocytes in the brain [57, 58]. Additionally, ApoE plays a neuroprotective role by activating synaptic formation and plasticity [44]. Further investigation is needed to determine whether the increase in ApoE protein following MSC administration differs according to *APOE* isoforms. CysC is a cysteine protease inhibitor found in the brain tissue [46]. Numerous studies have confirmed that CysC levels in the CSF and serum of patients with AD are lower than those in controls [59, 60]. CysC inhibits neuropathogenic cathepsins, binds to Aβ, and prevents Aβ oligomerization, fibril formation, and amyloid deposition [47]. These biomarkers may monitor MSCs responses for future MSC clinical trials for AD as a supplement to conventional AD biomarkers. In addition, these biomarkers may help understand the mechanism of action of MSCs in AD environment.

Functional analysis implied that MSCs are involved in neuronal development. In typical GR, gene ontologies associated with the nervous system were enriched, including positive regulation of synaptic assembly and nervous system development. Similarly, canonical pathway analysis demonstrated that the synaptogenesis signaling pathway was upregulated, indicating that MSC administration led to neuroprotective action in the typical GR. These results show the possibility that the GR group could ameliorate nervous system dysfunction in an AD environment via the increase in proteins associated with synaptogenesis after MSC administration. Additionally, in biological process, cell adhesion-associated genes were enriched, which may have increased MSC survival [61]. These results correspond with previous research, indicating that MSCs in AD CSF increase paracrine activity [9, 10].

The inflammatory response is predicted to appear after MSC administration due to the secretion of inflammatory cytokines, such as IL-15. Although MSCs are known to express major histocompatibility complex class I at a low level and rarely express class II, fever occurred within 36 h of administration of MSCs in the clinical trial *NCT02054208* [22]. Canonical pathway analysis revealed that the IL-15 production pathway is activated. Although the IL-15 production pathway was not identified in the typical PR, four proteins (major prion protein, retinoic acid receptor responder protein, protein kinase C-binding protein NELL1, and α-synuclein) that were associated with inflammation were identified by LC–MS/MS with FDR *q* < 0.05. In a previous study on the immune response to wild-type mouse MSC administration, the allogeneic group demonstrated significantly higher immune cell expression at the injection site than the syngeneic group [62]. An immune response may have occurred in the *NCT02054208* clinical trial because the transplanted MSCs were not autologous. The possibility of an immune response induced by the MSC culture medium α-Minimum Essential Medium (α-MEM) can also be considered, but in a previous study, very few immune cells were identified at the injection site in the group administered with α-MEM [62].

There are several limitations to this study. First, in the LC–MS/MS only a small number of participants (*n* = 9) was analyzed and the identified biomarkers were not replicated in ELISA when all the participants (*n* = 34) were analyzed. However, we believe our LC–MS/MS results are valuable in that the results suggested potential biomarkers related to MSC response from the first randomized human clinical trial of MSCs in AD. Further studies are needed to validate our results before these biomarkers could be used in clinical practice. Second, as we analyzed proteins that were identified in all three sets of LC–MS/MS, we might have missed some proteins that could have been detected only in a certain group. Finally, our results do not determine whether the proteins increased after MSC administration was secreted by MSCs itself or by the recipients in response to MSCs. Further studies using animal models are required to verify human-derived proteins.

## Conclusion

This study suggested the potential biomarkers that can be used to predict or monitor the response to MSC administration in patients with AD. Although the precise mechanism remains unknown, increased proteins after the third MSC administration appear to be associated with a decrease in CSF T-tau and P-tau levels and synaptogenesis activation.

## Supplementary Information

** Additional file 1**: Preparation of human umbilical cord blood (hUCB)-derived mesenchymal stem cells (MSCs).

**Additional file 2: Figures S1 and S2**. Selected potential prediction and monitoring biomarkers candidates. ELISA validation data.

**Additional file 3: Table S1**. Gene ontology and canonical pathway analysis data.

## Data Availability

The mass spectrometry proteomics data have been deposited to the ProteomeXchange Consortium via the PRIDE partner repository (https://proteomecentral.proteomexchange.org/cgi/GetDataset) with the dataset identifier PXD042108.

## Abbreviations

AD: Alzheimer's disease
MSCs: Mesenchymal stem cells
T-tau: Total tau
P-tau: Phosphorylated tau
CSF: Cerebrospinal fluid
GR: Good responder
PR: Poor responder
LC–MS/MS: Liquid chromatography/tandem mass spectrometry
ELISA: Enzyme-linked immunosorbent assay
RCN3: Reticulocalbin-3
FSTL3: Follistatin-related protein3
SCRG: Scrapie-responsive protein1
NPDC1: Neural proliferation differentiation and control protein 1
ApoE: Apolipoprotein E
CysC: Cystatin-C
Aβ: Amyloid-beta
hUCB-MSCs: Human umbilical cord blood-derived MSCs
PET: Positron emission tomography
TMT: Tandem mass tag
FDR: False discovery rate
ADAS-cog: Alzheimer's Disease Assessment Scale-Cognitive Subscale
MMSE: Mini-Mental State Examination
SD: Standard deviation
aMCI: Amnestic mild cognitive impairment
FC: Fold change
IL-15: Interleukin-15
α-MEM: α-Minimum Essential Medium
