# Supplementary material for: Identification of potential biomarkers related to mesenchymal stem cell response in patients with Alzheimer’s disease
Source: Stem Cell Res Ther. 2023 Jul 19;14:178. doi: 10.1186/s13287-023-03410-8 (PMC10357744; doi:10.1186/s13287-023-03410-8)
Supplement: Supplementary file 1 — Additional file 1: Preparation of human umbilical cord blood (hUCB)-derived mesenchymal stem cells (MSCs). [file 13287_2023_3410_MOESM1_ESM.docx]

**Supplementary Material and Methods**

**1. Preparation of human umbilical cord blood (hUCB)-derived mesenchymal stem cells (MSCs)**

hUCB-MSCs production, cell quality control, and quality assurance were conducted in accordance with the guidelines of the Korea Good Manufacturing Practices by the MFDS in order to be used for clinical purposes. After receiving written informed consent from normal women who were in their full-term pregnancy, hUCB tissue was obtained. hUCB-MSCs were raised in 10% fetal bovine serum supplemented α-Minimum Essential Medium (α-MEM, Gibco/Life Technologies, Carlsbad, CA, USA). Using 10% dimethyl sulfoxide, cells were cryopreserved at 150 °C or lower. Frozen hUCB-MSCs were thawed, seeded, and cultured prior to intracerebroventricular administration. Five days after being seeded, the cells were harvested, washed several times to remove impurities such as fetal bovine serum and trypsin, and then re-suspended in the proper volume of phenol red-free α-MEM. As a result, the final drug product, NEUROSTEM®, did not include fetal bovine serum. The viability, phenotype, and presence of endotoxins, bacteria, and mycoplasma in hUCB-MSCs were examined. Then, a total volume of 2 mL from the final adjusted concentration of 150 million cells per 1 mL of phenol red-free MEM- α was prepared for administration into each patient. hUCB-MSCs had a 48-hour shelf life and were kept at 4–12 °C. According to flow cytometric analyses, the cells consistently expressed the surface antigens CD73, CD90, CD105, and CD166 but not CD45, CD14, or HLA-DR.
