# Supplementary material for: Identification of potential biomarkers related to mesenchymal stem cell response in patients with Alzheimer’s disease
Source: Stem Cell Res Ther. 2023 Jul 19;14:178. doi: 10.1186/s13287-023-03410-8 (PMC10357744; doi:10.1186/s13287-023-03410-8)
Supplement: Supplementary file 2 — Additional file 2: Figures S1 and S2. Selected potential prediction and monitoring biomarkers candidates. ELISA validation data. [file 13287_2023_3410_MOESM2_ESM.pdf]

Supplementary Figure 1.

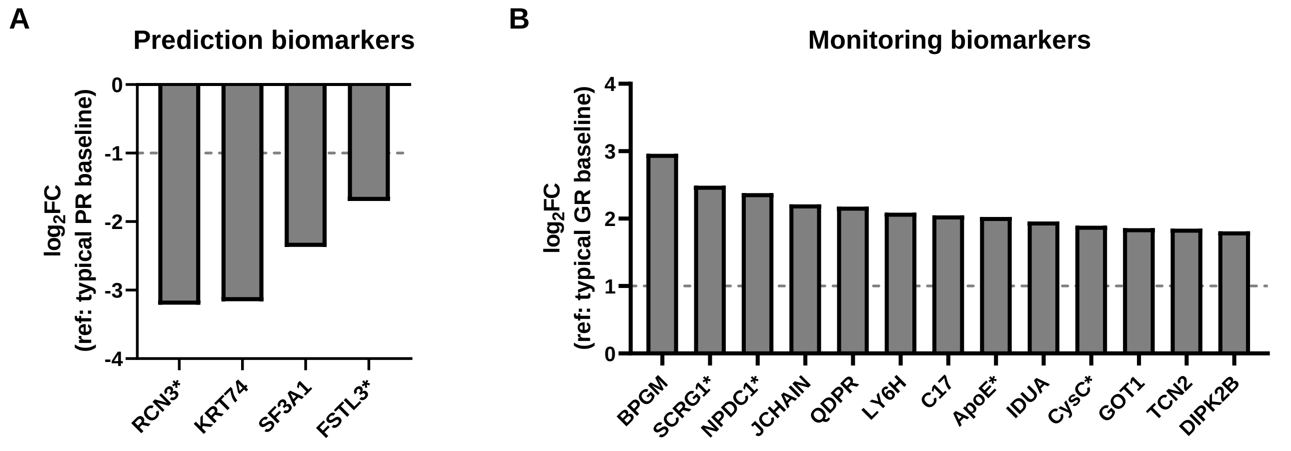

Supplementary Figure 1. (A) Four proteins were selected as prediction biomarker candidates that showed a significant difference in protein levels in baseline CSF between typical GR and typical PR (FDR q < 0.05, $|\log_2 FC| \geq 1.5$, unique peptide ≥ 2, Fold change = baseline protein levels of typical GR/PR). (B) 13 proteins were selected as monitoring biomarker candidates that showed significant change after the third injection compared to the baseline exclusively in typical GR (FDR q < 0.05, $|\log_2 FC| \geq 1.8$, unique peptide ≥ 2, Fold change = protein levels at one day after the third injection/baseline). *, Final selection of potential biomarkers based on their significance levels and known function. GR, Good Responder; PR, Poor Responder; FC, Fold change

Supplementary Figure 2.

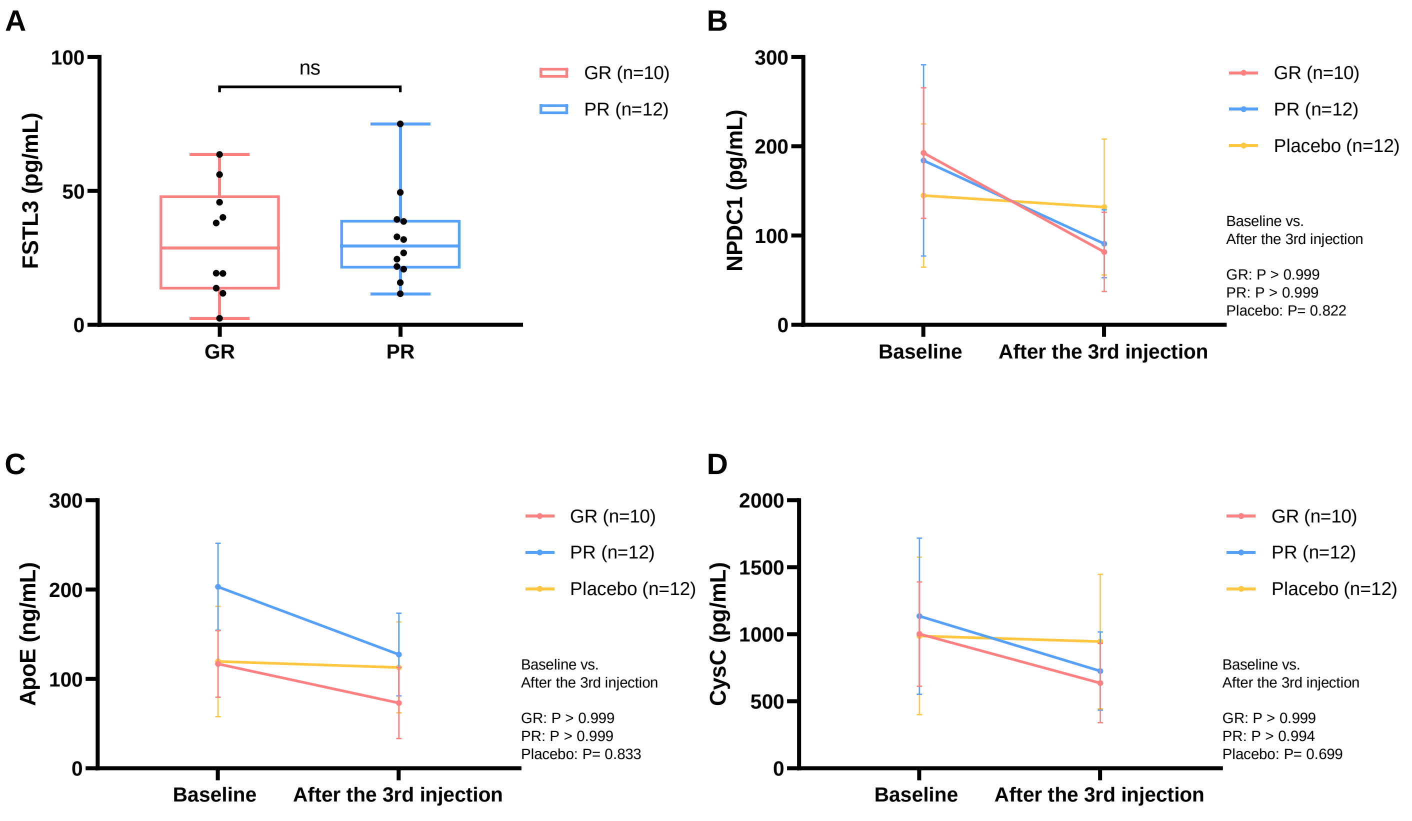

Supplementary Figure 2. Validation of potential prediction (FSTL3) and monitoring (NPDC1, ApoE, and CysC) biomarkers in all participants (n=34) via ELISA. Student's t-test or paired t-test (one-tailed) was used. ns; not significant, GR; Good Responder, PR; Poor Responder.
